# Factors affecting clinical outcomes in the management of melioidosis in Singapore: a 16-year case series

**DOI:** 10.1186/s12879-018-3393-1

**Published:** 2018-09-26

**Authors:** Jaime Mei-Fong Chien, Seyed Ehsan Saffari, Ai-Ling Tan, Thuan-Tong Tan

**Affiliations:** 1 Department of Infectious Diseases, Singapore General Hospital, Academia, 20 College Road, Level 3, Singapore, 169856 Singapore; 2 Centre for Quantitative Medicine, Duke-NUS Medical School, Academia, 20 College Road, Level 6, Singapore, 169856 Singapore; 3 Department of Pathology, Singapore General Hospital, Academia, 20 College Road, Level 3, Singapore, 169856 Singapore

**Keywords:** Melioidosis, *B. pseudomallei*, Bacteremia, Mortality, Recurrent, Singapore

## Abstract

**Background:**

*Burkholderia pseudomallei* is a gram negative bacteria that causes a spectrum of human diseases in the tropics. Although melioidosis is endemic in Southeast Asia, large clinical case series were rarely reported from metropolitan Singapore.

**Methods:**

This is a retrospective study of 219 consecutive patients with culture proven infections due to *Burkholderia pseudomallei* between the years 2001 to 2016 managed in Singapore General Hospital (SGH). We aimed to review local patients’ characteristics and identify clinical factors associated with mortality and recurrent melioidosis.

**Results:**

Culture proven melioidosis occurred in 219 patients, 83.1% were male with a mean age of 55.7 ± 14.3 years and 63.0% had diabetes mellitus. Most patients (71.7%) present within 4 weeks of symptom onset and the most common symptom was fever. The majority of patients had bacteremia (67.6%) and had infection involving the respiratory system (71.2%), presenting most frequently with multi-lobar pneumonia. Thirty-four (15.5%) deaths occurred during the initial hospitalisation with a median time from presentation to death of 6.0 days (interquartile range: 2.8–16.3). Twelve patients demised before the diagnosis of melioidosis was made. Univariate analysis identified patients with symptom duration of longer than 4 weeks, bacteremia, and disease requiring mechanical ventilation, inotropic support or temporary dialysis as factors that were significantly associated with mortality. Having bacteremia and disease requiring mechanical ventilation remained statistically significant factors in the multivariable analysis. Twenty-one (11.4%) patients developed at least 1 episode of culture proven recurrent infection, with 15 recurring within the first 12 months of their initial infection. Eight patients developed more than 1 episode of culture proven recurrent infection. Patients with multifocal infection were more likely to develop recurrent infection.

**Conclusion:**

In metropolitan Singapore, melioidosis was associated with mortality in excess of 15%, where more than a third occurred before diagnosis. This study reminds local physicians that melioidosis is still a serious infection affecting local male diabetic patients and an important differential diagnosis in a patient presenting with severe multi-lobar pneumonia and septic shock. Recurrent infections occurred in 11.4% and the weight-based dosing of oral eradication antibiotics may improve the management of this disease locally.

## Background

Melioidosis is an infection caused by *Burkholderia pseudomallei*, a gram-negative bacteria found in soil and fresh water in eastern tropical countries. Although notorious for its potential for bioterrorism, *B. pseudomallei* is a familiar cause of severe community acquired infection in endemic regions. Most cases were reported from northern Australia, Thailand and countries in Southeast Asia, and recently, there are increasing reports of cases worldwide [1–3]. A recent evidence-based predictive model suggested that human melioidosis may be underreported with an estimated global distribution of 165,000 cases per year with mortality in half of these cases [4]. The transmission of infection is predominantly via inhalation of the bacteria, which is postulated to come from aerosolization of soil and dust due to windy, rainy or flooding conditions. Infection may also be acquired via direct inoculation, aspiration and ingestion of the bacteria.

Singapore is a small metropolitan country with an area of 719 km^2^ and densely populated with a total population of 5.6 million people or 7797 persons per km^2^ [5]. People reside in high rise buildings and the country is well connected internally by various modes of transport. Singapore is a renowned hub for travellers on their transit. Due to her geographical location, Singapore is warm and humid throughout the year [6]. Despite the highly urbanised lifestyle people lead and the geographical shelter nature provides, Singapore continues to see new cases of melioidosis since it was first reported locally in 1920 [7]. Epidemiological studies found that in Singapore, where direct contact with soil is infrequent, melioidosis cases are associated with heavy rainfall 7 days preceding the onset of disease and to a lesser degree related to high humidity levels [8]. The surge in local cases noted in the year 2004 was correlated with annual rainfall and findings were supportive of these observations [7, 8].

Singapore General Hospital (SGH) is the largest adult tertiary teaching hospital with 1700 beds, situated in the south central region of the country and SGH manages approximately a quarter of the country’s new melioidosis cases annually. The aim of our study was to review the patients with culture proven melioidosis managed in SGH in the last 16 years and look for relevant demographical, clinical and management features in the hope to identify clinical pearls that will be useful to the local clinician. We also aimed to look for factors that may influence the patient’s inpatient mortality and development of recurrent infection in those who survived the initial presentation.

## Methods

We retrospectively reviewed the case records of patients treated in SGH with a positive culture for *B. pseudomallei* during the period 1 Jan 2001 to 31 Dec 2016. The demographical, clinical presentation and course of disease, treatment and outcomes were analysed. Patients who demised in the initial hospitalisation were compared to the patients who survived the initial hospitalisation. Patients who survived the initial hospitalisation and developed recurrent infections were further analysed.

In our institution, microbiological cultures were obtained by inoculating clinical specimens onto Trypticase Soy agar with 5% sheep blood and MacConkey agar and incubated at 35 degrees C for up to 48 h. For blood cultures, blood was inoculated into BACTEC/ F Plus Aerobic media and BACTEC/ F Plus Anaerobic media, and incubated in the BACTEC 9260 (prior to the year 2013) and BACTEC FX (from the year 2013 onwards) machines at 35 degrees Celsius for up to 5 days. Bottles flagged positive were cultured onto Trypticase Soy agar with 5% sheep blood, and MacConkey agar, and incubated at 35 degrees Celsius for up to 48 h. The bacteria were identified using API 20NE (bioMerieux, Lyon, France). Antimicrobial susceptibility of *B. pseudomallei* isolates were determined by disk diffusion test prior to August 2010 and by Etest thereafter.

### Definitions

Anti-melioidosis antibiotics were defined as the use of ceftazidime, imipenem or meropenem, trimethoprim & sulfamethoxazole (TMP-SMX), doxycycline, chloramphenicol or amoxicillin-clavulanate (Amoxi-Clav). We defined the completion of treatment as the receipt of at least 10 days of parenteral standard antibiotics as induction therapy and at least 12 weeks of eradication therapy. The time to death was calculated from the date of initial presentation to the date of death. Recurrent infection was defined in a patient who was on oral eradication therapy and showed an initial clinical and microbiological response to therapy and developed new symptoms and signs of infection in association with a culture positive for *B. pseudomallei*. The time between the initial infection episode and the recurrent episode was measured from the start of the oral eradication therapy to the onset of the first recurrent culture-confirmed infection.

### Statistical analysis

Statistical analysis was performed using SAS version 9.4 for Windows (SAS, Inc., Cary, NC USA). Statistical significance was set at *p* ≤ 0.05. Descriptive analysis—frequencies (percentages) for categorical variables and mean/median (standard deviation/inter-quartiles) for continuous ones—of clinical and structural characteristics were reported. Univariate logistic regression analysis was used to assess the association between the demographics and clinical features with the survival outcome. Multivariable logistic regression analysis was performed to adjust the odds ratios (OR) using stepwise selection criteria. We also performed both univariate and multivariable logistic regression analysis to investigate association between the baseline demographics and medical conditions with recurrent infections among those who survived.

## Results

There were 219 consecutive patients with culture proven first episode melioidosis during the study period. One hundred and eighty-two patients (83.1%) were male with a mean age of 55.7 ± 14.3 years. Almost all the patients (95.9%) were Singapore residents and the majority of patients (95.4%) had at least one comorbid condition at initial presentation. Diabetes mellitus was the most common pre-existing illness (63.0%), followed by hypertension (44.3%) and hyperlipidemia (30.6%) (Table 1). Most patients (71.7%) complained of acute symptoms with duration less than 4 weeks. The common symptoms complained were of fever (81.3%), cough (50.7%), chills (49.8%) and lethargy (48.4%).

**Table 1:** Demographic variables and baseline clinical features (*n* = 219)

Variable	Frequency (%) / Mean ± SD
Demographics	
Gender	
Male	182 (83.1)
Female	37 (16.9)
Age (years)	55.7 ± 14.3
Chinese	141 (64.4)
Singapore resident	210 (95.9)
Smoker	97 (44.3)
Comorbidities	
Diabetes mellitus	138 (63)
Hypertension	97 (44.3)
Hyperlipidemia	67 (30.6)
Ischemic heart disease	39 (17.8)
Lung disease	17 (7.8)
Liver disease	11 (5)
Renal disease	31 (14.2)
Malignancies	22 (10)
Rheumatological disease	12 (5.5)
HIV infection	1 (0.5)
Haematological condition	15 (6.8)
Stroke	11 (5)
History of tuberculosis	16 (7.3)
History of melioidosis	11 (5)
Immunosuppressive drugs	7 (3.2)
Symptoms	
Fever	178 (81.3)
Chills or rigors	109 (49.8)
Lethargy	106 (48.4)
Cough	111 (50.7)
Weight loss	62 (28.3)
Urinary symptoms	43 (19.6)
Joint pain or swelling	23 (10.5)
Total Duration of symptoms	
Less than 4 weeks	157 (71.7)
Disease Severity	
Bacteremia	148 (67.6)
Multifocal infection	109 (49.8)
Shock requiring inotropic support	46 (21)
Acute kidney injury requiring dialysis	24 (11)
Mechanical ventilation	47 (21.5)
Organ involvement	
Endovascular system	4 (1.8)
Lung	156 (71.2)
Liver	29 (13.2)
Splenic	42 (19.2)
Liver and spleen	13 (5.9)
Genitourinary tract	52 (23.7)
Musculoskeletal or SSTI	68 (31.1)
Central nervous system	16 (7.3)
Lymphatic system	60 (27.4)

One hundred and forty-eight (67.6%) patients had bacteremia, 21.0% had septicemic shock requiring inotropic support, 11.0% had acute kidney injury requiring temporary renal replacement therapy and 21.5% had respiratory distress requiring mechanical ventilation. Nearly half (49.3%) of the patients had infection involving multiple organs and the respiratory system (71.2%) was the most commonly involved organ system with multi-lobar pneumonia being the most frequent presentation. Twenty-nine (13.2%) patients had isolated liver abscesses, 42 (19.2%) had isolated splenic abscesses and another 13 (5.9%) had hepatosplenic abscesses. The genitourinary tract was involved in 52 (23.7%) patients with the majority (33 patients) having prostatic abscesses. Infection involving the lymphatic system was present in 60 (27.4%) patients and involvement of musculoskeletal or skin and soft tissue system was present in 68 (31.1%) patients (Table 1). Only 4 patients had endovascular infection and all these patients had mycotic aneurysms. Although 17 patients had central nervous system involvement, most were due to septic encephalopathy, and only 1 patient had *B. pseudomallei* isolated from the cerebrospinal fluid. Excluding blood specimens, clinical samples taken from the respiratory tract, lymphatic tissue and genitourinary tract were the top 3 most frequent organ systems for isolation of *B. pseudomallei*. All bacteria isolates found in the patients’ first positive culture were susceptible to ceftazidime; all but 1 isolate were susceptible to imipenem; 216 (98.6%) isolates were susceptible to Amoxi-Clav; 211 (96.3%) were susceptible to tetracycline. One hundred and thirty-six (62.1%) isolates were susceptible to TMP-SMX. However, the TMP-SMX susceptibility for 160 *B. pseudomallei* isolates were performed with the disk diffusion method (prior to August 2010) and amongst those isolates; there were 84 (52.5%) susceptible, 75 (46.9%) resistant and 1 intermediate resistant. The TMP-SMX susceptibility of the remaining 59 isolates were tested with the Etest and 52 (88.1%) were susceptible and 7 (11.9%) were resistant. Lastly, amongst 157 available chloramphenicol susceptibility results, 153 (97.5%) isolates were susceptible.

Amongst 204 patients who received induction therapy, 140 (63.9%) received ceftazidime monotherapy, 12 (5.5%) received carbapenem monotherapy, while 52 (23.7%) received a combination of antibiotics. The majority (92.3%) of patients who received a combination of antibiotics were ceftazidime based and most frequently combined with TMP-SMX (38.5%) or doxycycline (34.6%). Amongst 176 patients who received at least 10 days of induction therapy, 50 (28.4%) received less than 4 weeks, while 126 (71.6%) received 4 weeks or longer of induction therapy. The dose of ceftazidime received was an average of 100 to 120 mg/kg/day and the dose was adjusted to renal function where necessary.

Amongst 168 patients who received eradication therapy, 122 (72.6%) received at least 12 weeks of antibiotics. Thirty-four patients received between 12 to 20 weeks, while 88 patients received 20 or more weeks of eradication therapy. The combination of oral TMP-SMX and doxycycline was the most frequently received (67.3%) therapy during the eradication phase. A total of 47 (28.0%) patients received a single agent for eradication therapy, with Amoxi-Clav given most frequently in 19 of these patients. The dose of TMP-SMX given was adjusted for renal impairment but not weight based and dosed up to a maximum of 160/800 mg TMP/SMX twice daily in patients with normal renal function and doxycycline was uniformly dosed at 100 mg twice daily.

The median duration of the initial hospitalisation stay was 23.0 days (interquartile range: 12.0–42.0). Thirty-four (15.5%) deaths occurred during the initial hospitalisation with a median time from presentation to death of 6.0 days (interquartile range: 2.75–16.3). Twelve patients succumbed to the disease before the diagnosis of melioidosis was made; 11 of these patients had bacteremia and only 5 patients received at least one dose of ceftazidime or carbapenem before their demise. Analysis of the baseline demographics and medical conditions amongst patients who survived compared to the patients who demised in the initial hospitalisation found that pre-existing renal conditions (OR 0.37; 95% CI 0.15–0.89; *p* = 0.026), presenting symptom duration of 4 weeks or longer (OR 0.12; 95% CI 0.02–0.66; *p* = 0.0148), having bacteremia (OR 0.13; 95% CI 0.03–0.49; *p* = 0.0025), septicaemic shock requiring inotropic support (OR 0.10; 95% CI 0.04–0.22; *p* < 0.0001), acute kidney injury requiring temporary dialysis (OR 0.10; 95% CI 0.04–0.26; p < 0.0001) and respiratory distress requiring mechanical ventilation (OR 0.07; 95% CI 0.03–0.17; p < 0.0001) were significantly associated with inpatient mortality. Patients with presenting symptom duration of 4 weeks or longer, had bacteremia and required mechanical ventilation were factors that remained statistically significant in the multivariable analysis (Table 2). Patients who had skin and soft tissue infection or abscesses within the musculoskeletal system were more likely to survive the initial hospitalisation (OR 4.89; 95% CI 1.55–15.5; *p* = 0.0069). All ten patients with skin and soft tissue infections without bacteremia or another organ involvement survived and had no evidence of recurrent infection. While the majority of these patients (7 patients) received surgery with or without a course of antibiotics, only 2 patients with isolated skin and soft tissue infection had completed standard antibiotics treatment.

**Table 2:** Univariate and Multivariable Analysis of Demographics and Clinical Parameters with Survival, Logistic Regression model

Variable	Un-Adjusted Odds Ratio (95% CI)	*P* value	Adjusted^a^ Odds Ratio (95% CI)	*P* value
Smoker	1.78 (0.83, 3.82)	0.1414	6.05 (1.11, 33.2)	0.038
Renal	0.37 (0.15, 0.89)	0.026		
Rheumatological disease	0.78 (0.18, 3.43)	0.7397	0.01 (0.001, 0.22)	0.0056
Breathlessness	0.20 (0.09, 0.44)	<.0001		
Nausea or vomiting	0.66 (0.28, 1.51)	0.3216	3.67 (0.52, 26.1)	0.1943
Total duration of symptoms (<4 vs > 4 weeks)	0.12 (0.02, 0.66)	0.0148	1.6 (1.28, 2)	<.0001
Inotropes	0.10 (0.04, 0.22)	<.0001		
Dialysis	0.10 (0.04, 0.26)	<.0001		
Mechanical ventilation	0.07 (0.03, 0.17)	<.0001	0.02 (0, 0.18)	0.0004
Bacteremia	0.13 (0.03, 0.49)	0.0025	0.02 (0, 0.25)	0.0017
Musculoskeletal or SSTI	4.89 (1.55, 15.5)	0.0069		

^a^ Stepwise selection criteria is used for multivariable logistic regression analysis

Amongst 185 patients who survived the initial hospitalisation, 21 (11.4%) developed at least one episode of culture confirmed recurrent infection. The median time to the first recurrent infection was 20.9 weeks (interquartile range: 11.5–56.5). Fifteen (71.4%) patients recurred within the first 12 months of their initial infection. Compared to patients without recurrent infection, patients with recurrent infection tend to present with multifocal disease (OR 3.07; 95% CI 1.11–8.50; *p* = 0.0306) and had intra-abdominal abscesses (OR 2.59; 95% CI 1.01–6.63; *p* = 0.0468) (Table 3). Amongst the 146 patients without recurrent infection and who had received oral eradication therapy during their first melioidosis episode, there were 47 patients with isolates that were resistant to TMP-SMX in vitro (41 tested by disk diffusion and 6 tested by Etest). Thirty-one (66.0%) patients received a TMP-SMX based eradication therapy for their first melioidosis episode. Similarly, amongst 21 patients with recurrent infections, there were 13 patients with *B. pseudomallei* isolates that were resistant to TMP-SMX in vitro (all tested by disk diffusion) and 8 (61.5%) patients were treated with TMP-SMX based eradiation therapy during their first melioidosis episode. In terms of the proportion of patients who had completed therapy for the first melioidosis episode, 108 (65.9%) patients without recurrent infection compared to 9 (42.9%) patients with recurrent infection had completed treatment for their first melioidosis episode.

**Table 3:** Univariate and Multivariable Analysis of Demographics and Clinical Parameters with Recurrence, Logistic Regression model

Variable	Un-Adjusted Odds Ratio (95% CI)	*P* value	Adjusted^a^ Odds Ratio (95% CI)	*P* value
Liver disease	5.49 (1.22, 24.6)	0.0263	5.55 (1.13, 27.3)	0.035
History of tuberculosis	2.78 (0.72, 10.7)	0.136	3.67 (0.89, 15.2)	0.0733
Abdominal pain	2.96 (1.04, 8.37)	0.0414	2.61 (0.87, 7.86)	0.0871
Foci of infection (single vs multifocal)	3.07 (1.11, 8.50)	0.0306	3.43 (1.19, 9.87)	0.0224
Total treatment in weeks	0.99 (0.95, 1.02)	0.4193	0.97 (0.93, 1.01)	0.111

^a^ Stepwise selection criteria is used for multivariable logistic regression analysis

All patients with recurrent infections were re-initiated on intravenous induction antibiotics and continued on eradication oral antibiotics for each episode of recurrent melioidosis. Thirteen (61.9%) patients developed only 1 episode of culture proven recurrent infection and amongst them; 3 patients demised due to the infection. Amongst all patients with recurrent infections, 8 (38.1%) patients developed more than 1 episode of culture proven infections. Four patients developed a second episode of recurrent infection and 1 patient demised while on eradication therapy for the recurrent infection (the cause of death was attributed to advance metastatic cancer). Amongst 4 patients who developed a third episode of recurrent infection, there were 2 deaths and in both cases, the cause of death was attributed to septicaemia due to melioidosis.

## Discussion

Singapore General Hospital (SGH) is the largest adult tertiary hospital, located in the South-central part of Singapore and managed 26.8% of the country’s new melioidosis cases during the study period [9, 10]. We saw a similar trend of increase in new melioidosis cases during the wetter months of July to October and during January to December and this is supportive of the hypothesis that transmission of melioidosis is related to a heavier rainfall [8]. We postulate that inhalation may still be a common route of acquiring the bacteria as our country is a garden city and soil exposure is likely a consistent factor that locals do not recognise. We note a reduction in the number of new melioidosis cases per year in the last 6 years of the study (8.33 cases per year) compared to the prior 10 years (16.9 cases per year), and a similar trend reported nationwide during the corresponding period. From our study, the patients with infections presenting during the years of 2001 to 2010 tend to be multifocal infections compared to those presenting during the years 2011 to 2016.

Patients with diabetes mellitus are at high risk of acquiring the infection and this risk factor was the most common pre-existing illness in our series. The lungs were the most frequent organ affected in melioidosis, which is consistent with the postulation, that inhalation was the main acquisition route of infection. Approximately a fifth of our patients had splenic abscesses, and in fact, melioidosis was one of the top microbiological etiology along with tuberculosis for isolated splenic abscesses locally [11]. From the literature, the splenic involvement in our patients seemed to be similar to a Thai study, which reported splenic abscesses in 20.9% of their patients [12]. Thirty-three (18.1%) of our male patients had prostatic abscesses and this is comparable with an Australian study, which reported prostatic abscesses in 20.4% of their cohort [13]. Interestingly, there were a small number of patients with isolated cutaneous melioidosis, where the majority did not receive standard antibiotics treatment and were cured of infection. Gibney et al. reported a large cohort of Australian patients with cutaneous melioidosis and found that this group of patients were likely to be younger, had few medical conditions and had better outcomes compared with other forms of melioidosis infection [14]. Our findings agree with the authors’ suggestion that patients with isolated cutaneous melioidosis may not require standard parenteral induction and prolong oral maintenance therapy. In fact, we postulate that surgical incision and drainage may suffice for a selected group of patients with isolated cutaneous melioidosis. Future randomised controlled studies may be able to define this group of patients who may not need antibiotics treatment.

In the univariate analysis, inpatient-mortality was significantly related to the presenting symptom duration of 4 weeks or longer, bacteraemia, septicaemic shock requiring inotropic support, need for temporary dialysis or mechanical ventilation. These findings add to prior knowledge as important factors affecting inpatient mortality. [12, 15] In our series, more than one-third of the inpatient-mortalities occurred prior to the diagnosis of melioidosis and they did not receive anti-melioidosis antibiotics, understating the need for early diagnosis and the need for local physicians to think of melioidosis and empirically treat for such infections.

Culture proven recurrent melioidosis occurred less frequently in Australian patients at 6–13% [16, 17] and more frequently in Thai patients at 10–23% [18, 19]. Previous studies used multilocus sequence typing and discovered that approximately 75% of recurrent melioidosis were due to a relapse of the initial *B. pseudomallei* strain [16, 20] and 89% of the relapse infections occurred within the first 12 months of the initial infective episode [20]. We found 11.4% culture proven recurrence rate in our case series. Although we do not have genotyping results, more than two-thirds of our culture proven recurrent melioidosis cases occurred within the first 12 months of the primary infection, suggesting that most of our recurrences were due to relapse of the initial bacteria strain. Limmathurotsakul et al. described factors for recurrent, particularly of relapse infections, including the presence of bacteraemia, multifocal disease, the use of antibiotics other than the standard TMP-SMX and doxycycline combination during maintenance therapy and a maintenance therapy duration of less than 8 weeks [18]. The factors associated with recurrence were less clear from our study. In our series, patients with pre-existing hepatic conditions (OR 5.49; 95% CI 1.22–24.6; *p* = 0.0263) and multifocal infections (OR 3.07; 95% CI 1.11–8.50; *p* = 0.0306) were more likely to develop recurrent infections in the multivariable analysis (Table 3).

Our group of recurrent patients were more likely to have received a ceftazidime-based combination induction therapy with TMP-SMX or doxycycline compared to the group of non-recurrent patients. This practice was seen more commonly in the patients with infections acquired before the year 2006 as local physicians favoured combination, ceftazidime-based antimicrobials and longer duration of induction therapy to treat patients with deep-seated infections. When the evidence suggested that the addition of TMP-SMX to ceftazidime during induction therapy did not affect the mortality of patients [21], these practices gradually went out of favour by the year 2010. The duration of induction therapy received was not significantly different between the patients with recurrent infections compared to the patients with non-recurrent infections. The majority of patients in both groups received more than 4 weeks of intravenous induction therapy; 69.2% amongst the recurrent group and 57.1% amongst the non-recurrent group. As the majority of patients with recurrent infections had multifocal infections and that prompted the use of combination induction therapy. We postulate that the use of combination induction therapy failed to eradicate the deeply sequestrated bacteria foci and hence contributing to the recrudescence of infection.

The combination of oral TMP-SMX and doxycycline was the conventional regimen for the oral eradication phase of melioidosis treatment [22] until the recent decade. 

The MERTH study showed that treatment with a weight-based dosing of TMP-SMX was non inferior compared to the conventional treatment with TMP-SMX and doxycycline [23]. The doses of TMP-SMX used in the MERTH study were considerably higher than the doses used in the conventional TMP-SMX combination with doxycycline. The patients treated with TMP-SMX alone had an improved rate of recurrence (3% vs 6%) due to better drug adherence and less adverse drug reactions [23], suggesting that weight-based dosing of TMP-SMX may be more clinically effective in eradicating *B. pseudomallei* compared to the combination of non-weight based dose of TMP-SMX and doxycycline. Moreover, a dated in-vitro study demonstrated antagonism when TMP-SMX was used with doxycycline [24]. Hence, the current choice for eradication therapy is a weight-based dosing of TMP-SMX alone. In our series, most patients were treated with the conventional combination of TMP-SMX and doxycycline [22, 25] and thus, the doses of TMP-SMX used were considerably lower by the current standards [26], this could contribute to our recurrence rates. There were only 2 recurrent melioidosis in the cases collected after the year 2010, which might be due to the physicians’ gradual adoption of weight-based TMP-SMX for the eradication phase in the recent years or a product of bias from a shorter period of observation. There was a change of laboratory method for testing TMP-SMX susceptibility of *B. pseudomallei* isolates in August 2010 resulting in the resistance rate of 46.9% when disk diffusion was the method used and 11.9% when Etest was used. TMP-SMX susceptibility testing with disk diffusion was found to correlate poorly with Etest [27, 28]. This could be due to the difficulty of interpreting endpoints in testing susceptibility of *B. pseudomallei* to TMP-SMX [28]. Interestingly, in vitro resistance to TMP-SMX did not change the majority of our physicians’ choice of TMP-SMX during the oral eradication therapy and there were no significant differences in the patients’ recurrence rates due to the use of in vitro resistant TMP-SMX. We were unable to provide comments on the influence of the duration of oral eradication therapy as a third of the recurrent patients defaulted and more than half of the remaining recurrent patients develop the first recurrent infection during the eradication phase of their first melioidosis episode.

Our study was limited by the retrospective methodology and subjected to recall bias and errors due to incomplete case records. Non-digitalised data was collected by a single investigator to minimise inter-observer differences. This is the largest clinical case series of melioidosis in Singapore with cases accumulated over the period when several practice-changing studies were published on the management of melioidosis.

## Conclusions

In metropolitan Singapore, melioidosis is still a serious infection affecting male, diabetic patients presenting with severe multi-lobar pneumonia and septic shock. Patients presenting with *B. pseudomallei* bacteremia and severe organ dysfunction were strongly associated with mortality. These factors should trigger a high clinical suspicion for melioidosis and prompt empirical anti-melioidosis treatment. The factors associated with recurrent infections were indistinct but we postulate that the gradual shift in clinical practice towards a higher dosing of TMP-SMX given during the eradication therapy may improve the rates of recurrent disease locally.

## Abbreviations

Amoxi-Clav: Amoxicillin-clavulanate
OR: Odds ratio
SGH: Singapore General Hospital
SSTI: Skin and soft tissue infection
TMP-SMX: Trimethoprim and sulfamethoxazole
